# Flexible, self-powered sensors for estimating human head kinematics relevant to concussions

**DOI:** 10.1038/s41598-022-12266-6

**Published:** 2022-06-23

**Authors:** Henry Dsouza, Juan Pastrana, José Figueroa, Ian Gonzalez-Afanador, Bianca M. Davila-Montero, Nelson Sepúlveda

**Affiliations:** 1 Department of Electrical and Computer Engineering, Michigan State University, East Lansing, MI USA; 2 Department of Mechanical Engineering, Michigan State University, East Lansing, MI USA

**Keywords:** Biophysics, Engineering, Materials science

## Abstract

The present work demonstrates the development of a flexible, self-powered sensor patch that can be used to estimate angular acceleration and angular velocity, which are two essential markers for predicting concussions. The device monitors the dynamic strain experienced by the neck through a thin, polypropylene-based ferroelectret nanogenerator that produces a voltage pulse with profile proportional to strain. The intrinsic property of this device to convert mechanical input to electrical output, along with its flexibility and $\sim$ 100 $\mu$m thickness makes it a viable and practical device to be used as a wearable patch for athletes in high-contact sports. After processing the dynamic behavior of the produced voltage, a correspondence between the electric signal profile and the measurements from accelerometers integrated inside a human head and neck substitute was found. This demonstrates the ability of obtaining an electronic signature that can be used to extract head kinematics during collision, and creates a marker that could be used to detect concussions. Unlike accelerometer-based current trends on concussion-detection systems, which rely on sensors integrated in the athlete’s helmet, the flexible patch attached to the neck would provide information on the dynamics of the head movement, thus eliminating the potential of false readings from helmet sliding or peak angular acceleration.

## Introduction

Collisions in high-contact sports have been studied for causing neuropsychological changes^1^ and neurodegenerative diseases^2^. One example is chronic traumatic encephalopathy (CTE), which is a progressive degenerative brain disease. It is a consequence of a repeated concussions and traumatic brain injuries, such as those experienced by athletes in high-contact sports. A brain with CTE gradually deteriorates and loses mass. The disease not only deteriorates skills necessary for daily life (e.g. memory, self-control, time-management, focus); but it also causes much bigger mental problems (e.g. strong suicidal thoughts, depression, cognitive and thinking problems, anxiety, violent/abusive behavior, dementia) which become worse over time. A report in 2017^3^ showed that 99% of National Football League (NFL) players (in United States of America) in the study had CTE. The study also showed that 91% of College Football, and 21% of high-school players in the study also had CTE.

However, significant brain damage can still occur without impact or collision. High-acceleration head movements; typically referred to as “whiplashes”, which can occur in non-contact sports, can also generate brain injuries such as concussions. The Center of Diseases Control and Prevention (CDC)^4^, defines a concussion as *a type of traumatic brain injury–or TBI–caused by a bump, blow, or jolt to the head or by a hit to the body that causes the head and brain to move rapidly back and forth.* This sudden movement can cause the brain to bounce around or twist in the skull, creating chemical changes in the brain and sometimes stretching and damaging brain cells. The CDC has reported that 20% of the estimated 1.7 million concussions per year are sports-related^5,6^. Research suggests that after a concussive event, the brain goes under metabolic crisis^7^. This metabolic disruption lasts about a week and any concussive event during this period leads to greater cognitive impairment which lasts for a longer time^8^. This is the main reason why an athlete who suffered from concussion is removed out of play and rested immediately.

To identify the severity of an impact and risk of a TBI, many criteria factors have been defined such as Wayne State Tolerance Curve (WSTC)^9^, Gadd Severity Index (GSI)^10^ and Head Injury Criteria (HIC)^11^. These measures were developed to quantify severe brain injuries caused by linear head acceleration^12^. However, because of the human head-neck anatomy, injuries only due to linear or translational acceleration are very rare. Translational and rotational acceleration need special conditions to occur as isolated events, therefore it is more common to observe incidents of angular acceleration^13^.Rotational kinematics have shown to cause major brain tissue damage due to strains^14^ and thus is a need of quantifying them in order to estimate the extent of TBIs.

**Figure 1:**
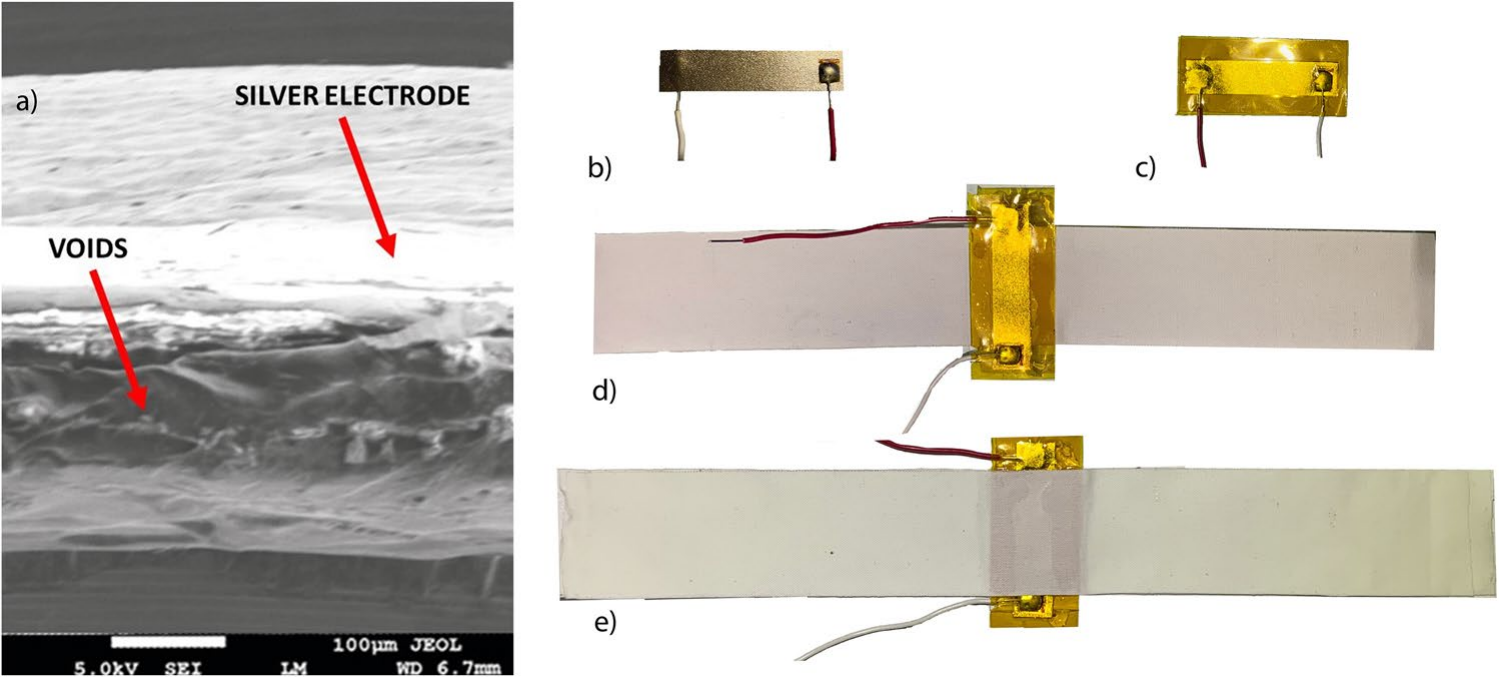
(**a**) SEM imgae of FENG’s cross-section, (**b**) The FENG after deposition of silver electrodes and fixing wire electrodes. (**c**) Encapsulation in Kapton tape to protect the electrodes. (**d**) A Thin layer of PDMS is placed on one side of the FENG and glued to the first kinesiology-tape (K-tape). (**e**) PDMS and K-tape placement is repeated on the opposite side of the FENG, resulting in a fully encapsulated device.

Head Impact Telemetry (HIT) System is a technology currently being used to measure and record in-vivo head impact exposure^15,16^. The system consists of an array of accelerometers that are physically connected to the helmet, and trigger data collection whenever any of the accelerometers measures an acceleration beyond certain threshold. This technology was first implemented in 2003^17^, and is currently being used in commercially available helmets. The main limitation of this approach is that it relies on sensors attached to the helmet – they are not directly in contact with the athlete’s head. As it has been pointed out by experts in biomechanics, the sliding that can occur between the helmet and the head makes it difficult to predict how the brain moves inside the skull^18^. A solution to this sliding problem was presented by the X-Patch, which had two versions: one used as a skin patch behind the ear, and the other in a mouth guard^19^. Both of these systems consist of accelerometers that track the rotational speed of the impact. However, the resonance of the micro-mechanical structures inside the MEMS-based accelerometers are within the range of head motion frequencies. This has been found to cause large errors when measuring the peak angular accelerations^20^. In this work, we present an alternative device that overcomes these obstacles, and could result in more accurate estimation of head kinematics. The device consists on a Ferro-Electret Nano-Generator (FENG), which is formed from a flexible, thin polypropylene (PP) piezoelectret film with micrometer-scale “quasi-dipoles” across its thickness and electrodes at both surfaces as shown in Fig. 1a. FENG devices have been used in the past for several energy harvesting and self-powered sensor applications^21–24^. Applying a mechanical stress reshapes the dipoles, generating charge accumulation in the electrodes, thus resulting in an electrical output in the form of an electric potential difference between the electrodes, or the flow of charge across a load connected between those electrodes (i.e., voltage or current)^25^. This phenomenon is commonly referred to as “quasi-piezoelectricity”, and these devices have been demonstrated to be useful in a wide variety of applications such as loudspeaker, microphone^26,27^, structural health monitoring^28^ and energy harvesting^29^.

**Figure 2:**
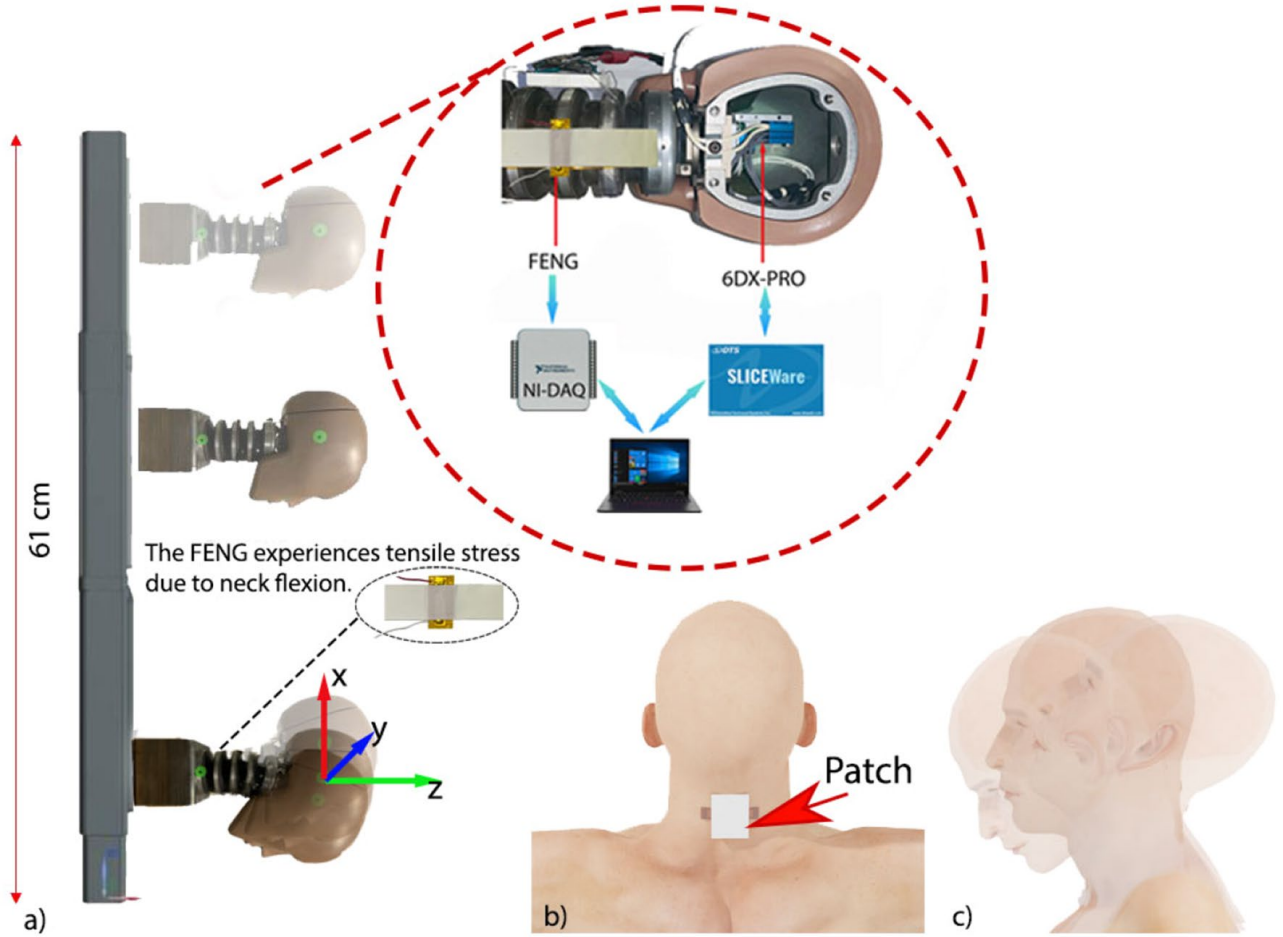
(**a**) A dummy is fixed to the center plate, then dropped in free-fall. The inset shows the position of 6DX accelerometers inside the dummy head, and the patch over the neck. (**b**) envisioned location of the patch during implementation, and (**c**) head movement/kinematics “whiplash” that can can be characterized using the developed patch.

In this work, a wearable patch is used to monitor and describe the tensile forces developed at the neck of a human head substitute (hereinafter referred to as “dummy”). The patch is placed on the back (shown in Fig. 2 and front (not shown) of the dummy neck and they experience tensile stress as neck expands. This stress produces a corresponding electrical output from each device. We analysed the relationship between these two dynamic parameters (neck strain and FENG’s electrical output), and developed a model to correlate the measured electrical pulse profile to the kinematic signature of a human head, with the goal of developing a more reliable concussion-detection system. Unlike other helmeted devices used on high-contact sports, where the sensors required to measure human head kinematics are placed inside the helmet, the sensor patches used in this work are placed directly on the neck region; which extends their use to sports that do not require the use of helmets, and eliminates false readings from helmet-only movements. In addition, FENG devices are self-powered sensors—i.e. they do not need an external electrical power source to operate, minimizing operation complications and safety hazards that other sensors can bring to an athlete.

## Results

### Experiment configuration

The testing setup consists of three main components: (i) drop towers (comprising of the rails and a center plate); (ii) head and neck assembly, and (iii) flexible FENG sensors. The drop tower rails are constructed using parallel telescopic tubes measuring approximately 60 cm. The center plate is welded only to the outer telescopic tubes such that it can move freely normal to the floor as shown in the setup described in the supplementary note. The second piece of the setup is a Hybrid III head and neck form assembly from Humanetics (Hybrid III 50th Male,Standard ATD 78051-218-H, hereinafter referred to as a “dummy” ), which is commonly used for crash test experiments^30,31^. This dummy emulates a $50^{th}$ percentile human body head that provides a mounting block for the triaxial accelerometer with integrated triaxial angular rate (DTS-6DX PRO; hereinafter referred to as “6DX”) sensor at its center of gravity, as shown in Fig. 2. The head and neck are mounted to the welded centre plate (as shown in the supplementary figure S2). The third component is the patch which is described in the following section.

As shown in Fig. 1a, after depositing silver electrodes, electrical leads were attached to the device (5 cm x 1 cm) followed by its encapsulation in Kapton tape to protect the electrodes(Fig. 1b). A thin layer of PDMS was placed on one side of the FENG, which was then glued to a therapeutic kinesiology tape (K-Tape) using epoxy. This was followed by placing PDMS and a second K-Tape on the opposite side of the FENG, resulting in a patch-like, fully encapsulated configuration as shown in Fig. 1c and d. This PDMS was fabricated by mixing the base agent and the curing agent in the ratio of 10:1, respectively; followed by curing at $100^{o}\hbox {C}$ for 90 mins. This ratio yields a film of young’s modulus $\approx$ 1.5MPa^32^ and the thickness of PDMS film is $\approx$ 1mm. This arrangement resulted in applied tensile force to the FENG device upon stretching of the tape, due to the changes in the neck’s radius of curvature during a fall. In a separate experiment, it was verified that during compression, buckling occurred only at the K-tape regions and the FENG did not experience significant stress i.e. the produced/measured voltage was only due to tensile stress and not compression (see supplementary note for further details). For simplicity, the encapsulation of the FENG device, including electrodes and tape, will be referred to as “the patch”.

**Figure 3:**
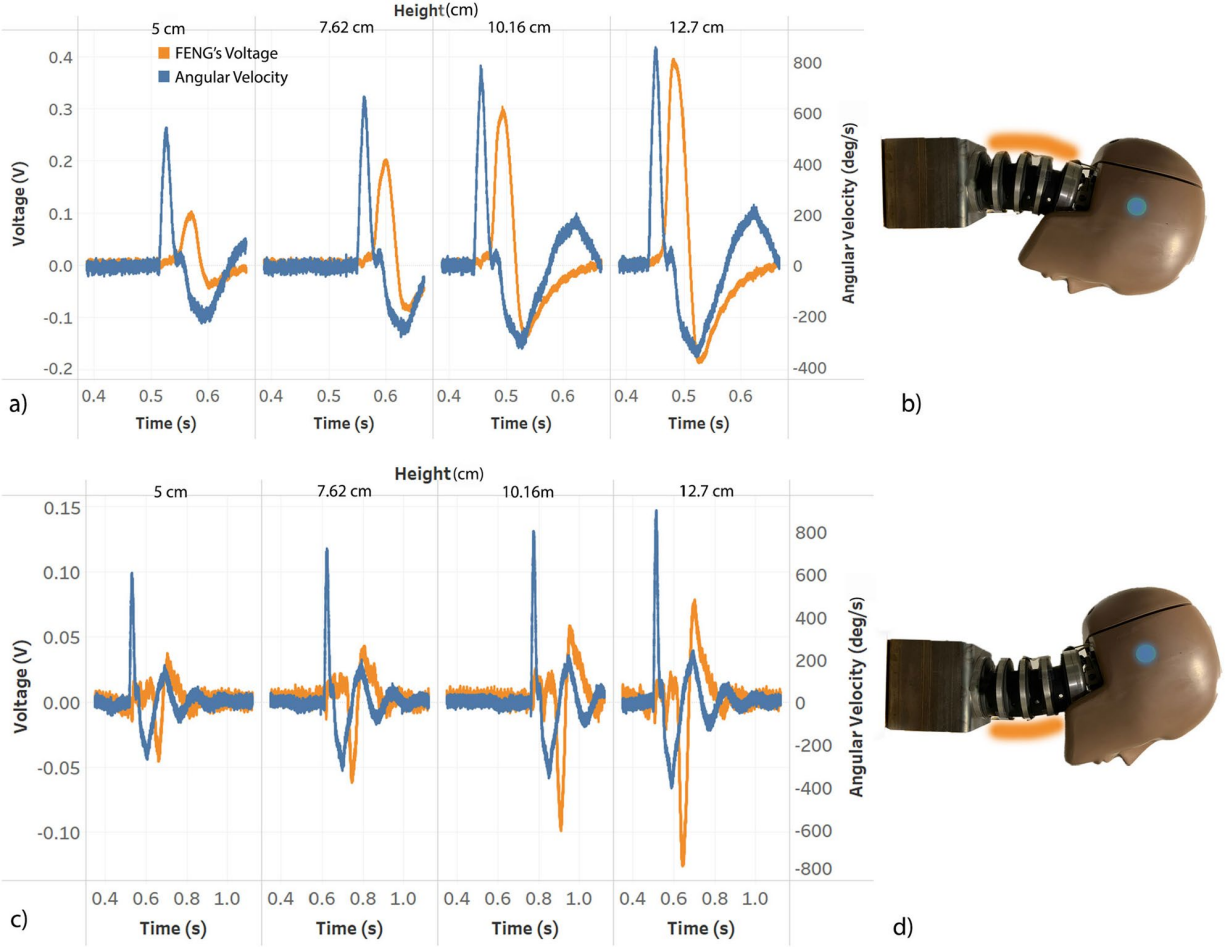
Measured responses to hyper-flexion and hyper-extension of the neck. (**a**) Hyperflexion: Voltage response of the patch located at the back of the neck (as shown in (**b**), where the orange shade represents the patch location) and angular velocity from the 6DX for drop tests from various heights simulating frontal collision of the center plate. (**c**) Hyperextension: Voltage response of the patch located at the front of the neck (as shown in d) and angular velocity from the 6DX for drop tests from various heights simulating frontal collision of the center plate. Blue dots on (**b**) and (**d**) are rough estimations of the 6DX position.

As shown in Fig. 2a (insert), the patch is placed on the back of the neck and held on by using clamps (not shown in the figure for clarity). These experiments correspond to the measurements shown in Fig. 3a and b. Similarly, the patch was placed in the front side of the neck for the measurements shown in Fig. 3c and d.

The movement of the neck in this work is restricted to rotational displacements around the “y” axis, following the axes shown in Fig. 2a. To reproduce this movement, the head is securely attached to the center plate, facing down the rails and dropped, with gravity being the only force acting in the process. This head motion is similar to the frontal crash in automobiles, where the head experiences a whiplash effect (both hyperflexion and hyperextension), as shown in Fig. 3b and d, and also in supplementary video 1. Translational movement along the x-axis occurs during the drop, and the rotation along the y-axis represents the whiplash, triggered when the translation movement around the x-axis ends. It should be noted that there is a very small hyperextension effect at the onset of the translation movement long the x-axis—i.e. when the drop action begins. However, this is insignificant when compared to the whiplash at the end of the translation movement. This whiplash effect can also be observed in high-contact sports like judo, wrestling, and football when a player is suddenly moved or pushed by an opponent in the chest area, causing the head to experience a sudden angular acceleration. It must be noted that there is no direct collision to the head during the experiments—only whiplash. Although most of the applications/systems demonstrated with FENG devices use a mechanical input normal to the electrode surface (i.e. in- and out-of plane motion), their generated voltage pulse profile is produced by the compression/relaxation of the internal dipoles, which can be obtained through tensile mechanical input^28^.

### Signal acquisition, conditioning and processing

The 6DX is configured in “recorder mode” (i.e., triggered by a pulse signal), and the data is recorded using SLICEWARE (Software by DTS for interfacing with the sensor). The data from the patch is recorded using a data acquisition system from National Instruments (NI-DAQ 6003) and, similar to the 6DX, the recording was also triggered with the same pulse signal of the 6DX. By using a double pole double throw switch, the 6DX and NI-DAQ are triggered simultaneously. The unfiltered data from both is stored for 2 *s* after the triggering action, and is shown in Fig. 3. Figure 3a shows the results for the output of the back neck area patch, while Fig. 3b shows the measured output from the front neck area patch. In both figures, a delay between the FENG response and the angular velocity data from 6DX is observed. This is due to the position of the sensor relative to the FENG. The head neck linkage acts like a damper and also the neck flexion by itself has an damped behaviour. This causes the FENG’s response which is placed on the neck to lag behind the 6DX sensor response which is placed in the head.

**Figure 4:**
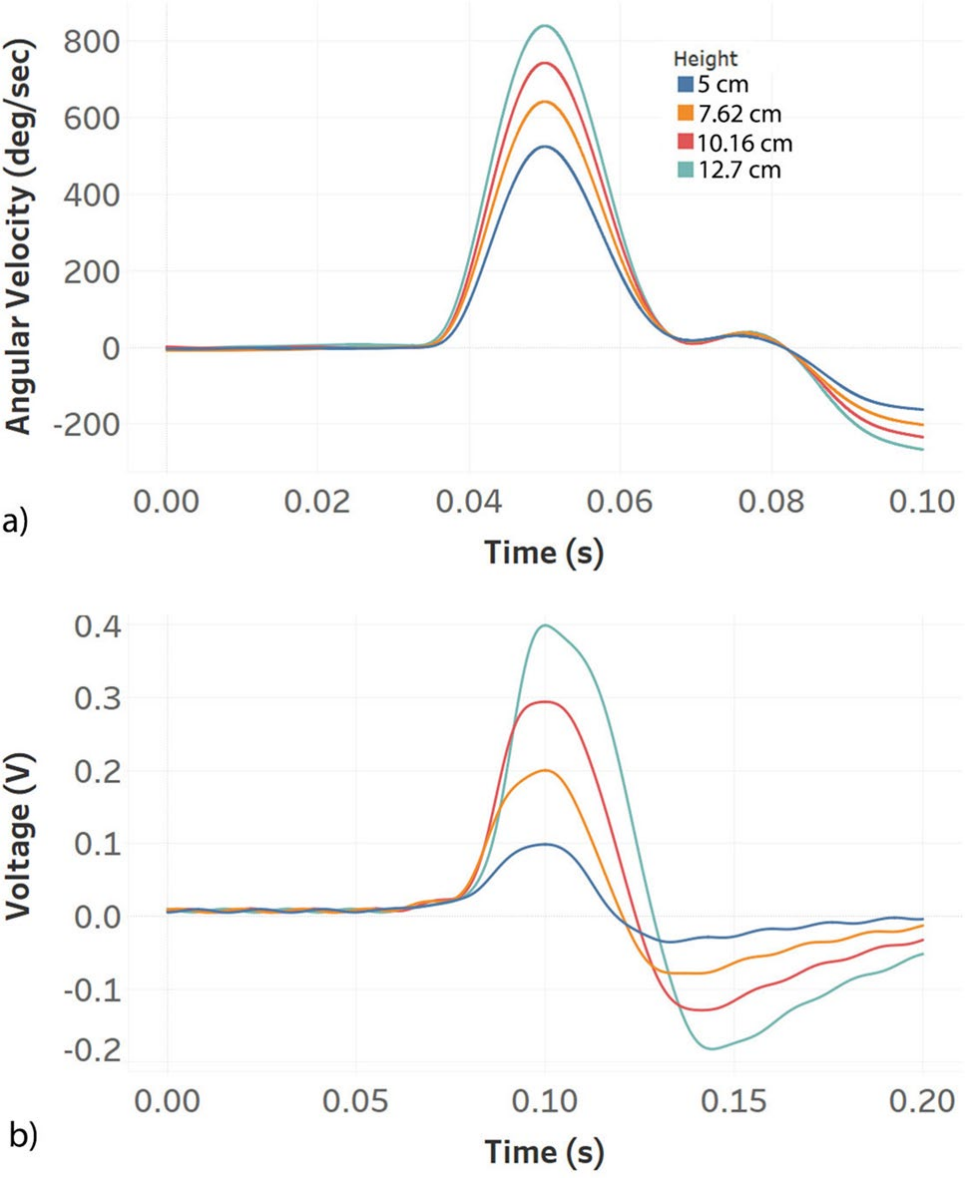
For different drop heights (**a**) filtered angular velocity and (**b**) filtered voltage.

The raw signals were filtered through a fourth order Butterworth low-pass filter with a cut-off frequency of 100 Hz, which is one of the standard filters in the SAE standard^33^. In most cases this cut-off frequency will attenuate signals generated from direct impact on the FENG since these events are quicker compared to the flexion of the neck. Given that the voltage peak response from the patch can be above the input limits of the NI-DAQ system (+ 10 V), the voltage output is attenuated through a resistive voltage divider and finally fed to the DAQ. It is important to note that the impedance seen by the FENG influences its dynamic performance^29^. In this case, the net load seen by the patch is 2.5 G$\Omega$ and this impedance was found to produce accurate voltage output pulse profiles^28^. The sampling rate for both sensors is set to 50 kHz, and the data is further processed using Python software^34^. Both filtered signals are shown in Fig. 4

### Modeling

**Figure 5:**
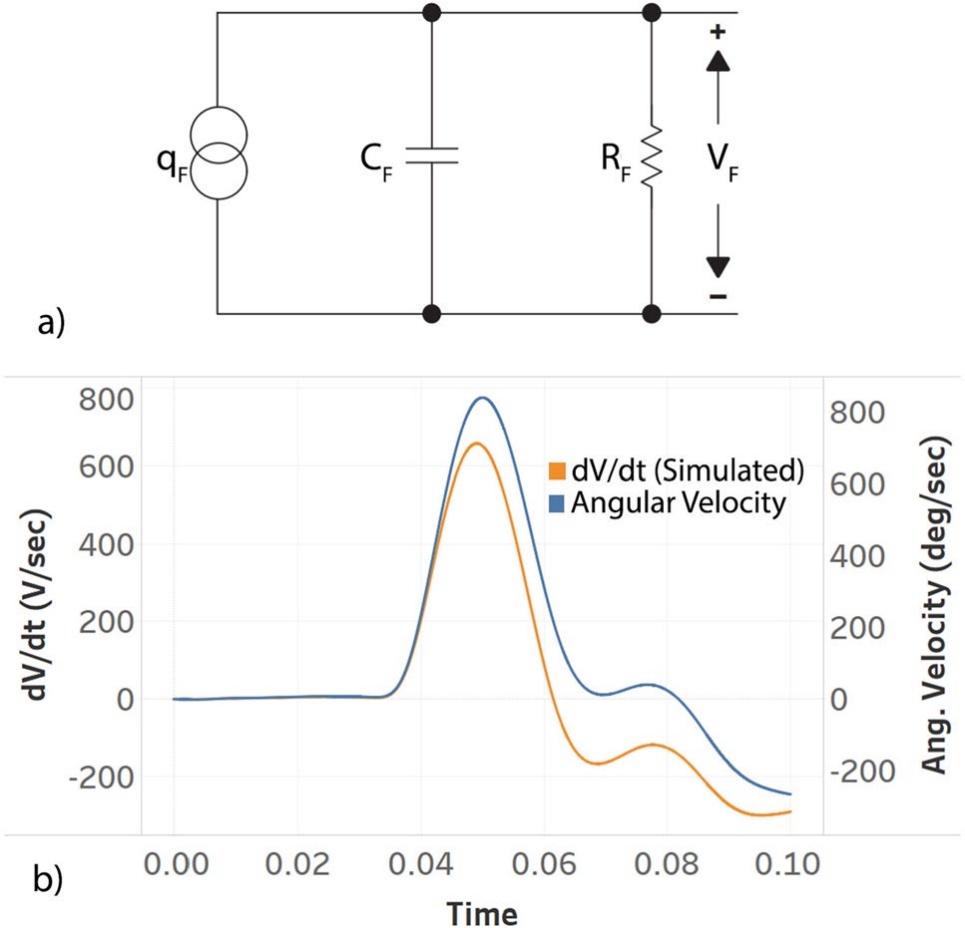
(**a**) Equivalent circuit model of the FENG. (**b**) Angular velocity and simulated first derivative of the FENG’s voltage plotted on a dual y-axis.

Considering the FENG device as a flexible piezoelectric, it can be modeled as a charge source ($q_F$) with a shunt capacitor ($C_F$) and resistor ($R_F$) as shown in Fig. 5. The charge produced depends on the piezoelectric coefficient. The capacitance is defined by the thickness, cross-sectional area and the dielectric constant of the material. The resistance represents the dissipation of the accumulated charge. The transfer function for this circuit is given by equation (1a). Based on piezoelectric properties, the charge is proportional to an applied force (with the piezoelectric coefficient being constant). From the position of the FENG relative to the 6DX sensor as shown in supplementary figure S3, it can be inferred that the force experienced (tensile) by the FENG is proportional to the angular position of the 6DX (1b), thus making the angular velocity proportional to rate of change of force (1c).

From the previous analysis, we can argue that the transfer function between the rate of change of voltage and angular velocity should be similar to that of (1a), which is represented in (1d). The value of $R_F$ can be estimated based on the result from Cao et. al^29^. The intrinsic resistance of the FENG is $\ge 600 M\Omega$ and this is in parallel to net impedance provided by the voltage divider and input impedance of the NI-DAQ. Thus $R_F$ is $\approx 550 M\Omega$, and the capacitance to be of the order of 100 pF.

This result was confirmed by analytical computer-based simulations of the transfer function with the angular velocity recorded from the 6DX, and comparing the results with the generated rate of change of voltage. A representative instance of such correlation between the simulated and measured signals is shown in Fig. 5b –it should be noted that the scale of the voltage signal is not relevant for the correlation, since the emphasis is placed on a dynamic correspondence that allows for mapping between plots. This confirms the hypothesis that the first derivative of the FENG’s voltage can be mapped to angular velocity, as long as the proportionality constant is known.

1a $$\begin{aligned}&\frac{V_F(s)}{q_F(s)} = \frac{sR_F}{1+sC_FR_F} \end{aligned}$$

1b $$\begin{aligned}&q_F \propto F(Force) \propto \theta (Angular\,Position) \end{aligned}$$

1c $$\begin{aligned}&\frac{dF}{dt} \propto \omega (Angular Velocity) \end{aligned}$$

1d $$\begin{aligned}&\frac{\dot{V}_F(s)}{\omega (s)} \propto \frac{sR_F}{1+sC_FR_F} \end{aligned}$$

**Figure 6:**
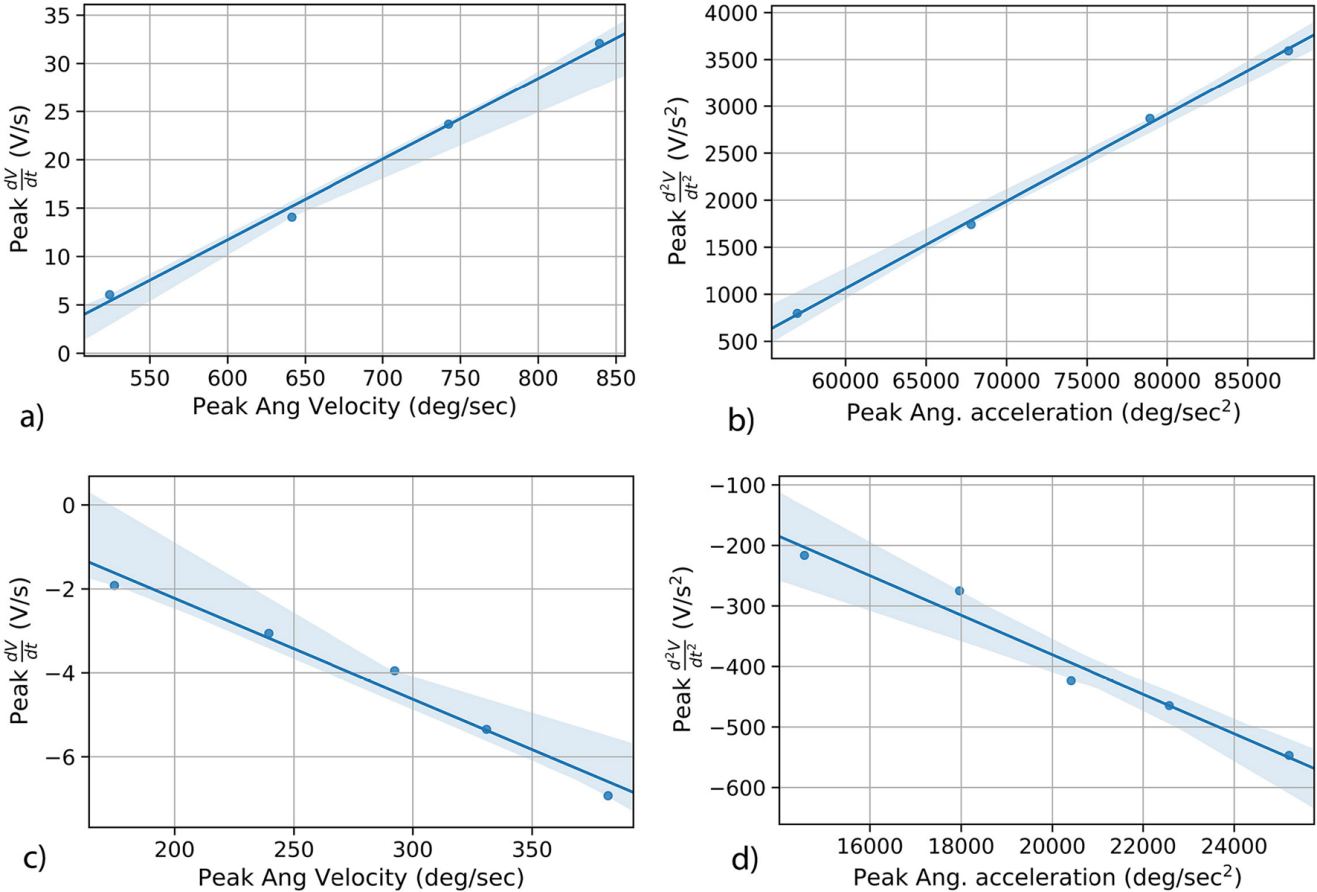
(**a**) and (**c**) relationship between the FENG’s voltage response and the peak angular velocity during hyperflexion and hyperextension. Similarly, (**b**) and (**d**) show the relationship between FENG’s voltage response and the peak angular acceleration. The shaded region shows the 95 % confidence interval of the fitted line.

### Estimating head kinematics from FENG response

As shown in Fig. 6, the aforementioned features show a strong linear correlation with the FENG’s response. Figure 6a and b show that, during hyperflexion, the peak angular velocity and acceleration can be determined by knowing the peak $\frac{dV}{dt}$ and peak $\frac{d^2V}{dt^2}$ from the FENG’s response; the FENG’s output voltage by itself is a measure of strain on the neck during these events. A similar behaviour is seen when the patch is placed on the front side of the neck to determine the markers due to hyperextension.

The results indicate that it is possible to estimate the head rotational kinematics by simultaneously recording signals from both patches. This is in part possible due to their construction and location. This is supported by closely observing Fig. 3c: when placed on the front of the neck, the patch produces a negligible voltage response during hyper flexion, but produces a significant response during hyper extension.

## Conclusion

This work shows that there is an strong positive correlation ($R^2 > 90\%$) between the patch output and rotational kinematic signatures experienced by the human head, which was recorded using a triaxial accelerometer and gyroscope placed at the center of gravity of the human head dummy. This result is also supported by the proposed model which yields a response similar to the one measured by the patch. The FENG which at the heart of this patch has $<10\%$ variation in its sensitivity across devices. Although this work demonstrates the system along one spatial axis, it can certainly be expanded along the other axes by placing multiple sensors around the neck; thus providing a full, comprehensive map of the human head during a collision. The risk of false positive signals originating from direct impact on the FENG can be mitigated through appropriate filtration schemes as mentioned earlier and also by reducing the surface area of the sensor which will make it less likely to experience accidental direct impact. Even in the lack of software filter the FENG’s load can be modified to change its frequency response.

## Supplementary Information

Supplementary Information 1.

Supplementary Video 1.

## References

[1] Hebb DO (2005). The organization of behavior: A neuropsychological theory.

[2] Barnham KJ, Masters CL, Bush AI (2004). Neurodegenerative diseases and oxidative stress. Nat. Rev. Drug Discov..

[3] Mez J, Daneshvar DH, Kieman PT (2017). Clinicopathological evaluation of chronic traumatic encephalopathy in players of american football. JAMA.

[4] What is a concussion? (2019). https://www.cdc.gov/headsup/basics/concussion_whatis.html.

[5] Faul, M., Wald, M. M., Xu, L. & Coronado, V. G. Traumatic brain injury in the united states; emergency department visits, hospitalizations, and deaths, 2002-2006. *CDC* (2010).

[6] Report to congress on mild traumatic brain injury in the united states: steps to prevent a serious public health problem. *Atlanta, GA: Centers for Disease Control and Prevention* **45** (2003).

[7] Giza CC, Hovda DA (2014). The new neurometabolic cascade of concussion. Neurosurgery.

[8] Vagnozzi R (2008). Temporal window of metabolic brain vulnerability to concussion: A pilot 1h-magnetic resonance spectroscopic study in concussed athletes–part iii. Neurosurgery.

[9] Gurdjian, E. S., Lange, W. A., Patrick, L. M. & Thomas, M. E. Impact injury and crash protection (1970).

[10] Gadd, C. W. Use of a weighted-impulse criterion for estimating injury hazard. Tech. Rep., SAE Technical Paper (1966).

[11] Eppinger, R., Kuppa, S., Saul, R. & Sun, E. Supplement: development of improved injury criteria for the assessment of advanced automotive restraint systems: Ii. *NHTSA* (2000).

[12] Greenwald RM, Gwin JT, Chu JJ, Crisco JJ (2008). Head impact severity measures for evaluating mild traumatic brain injury risk exposure. Neurosurgery.

[13] Kleiven S (2013). Why most traumatic brain injuries are not caused by linear acceleration but skull fractures are. Front. Bioeng. Biotechnol..

[14] Meaney DF, Olvey SE, Gennarelli TA (2011). Biomechanical basis of traumatic brain injury. Youmans Neurol. Surg..

[15] Beckwith JG, Greenwald RM, Chu JJ (2012). Measuring head kinematics in football: Correlation between the head impact telemetry system and hybrid iii headform. Ann. Biomed. Eng..

[16] Crisco JJ, Chu JJ, Greenwald RM (2005). An algorithm for estimating acceleration magnitude and impact location using multiple nonorthogonal single-axis accelerometers. J. Biomech. Eng..

[17] Duma, S. M. *et al.* Analysis of real-time head accelerations in collegiate football players (2005). 10.1097/00042752-200501000-00002 15654184

[18] Moore, N. C. Understanding concussions: Testing head-impact sensors. https://news.umich.edu/understanding-concussions-testing-head-impact-sensors/.

[19] Rains, B. X2 biosystems introduces their next generation x-patch pro head impact monitor (2016).

[20] Camarillo, D. B., Shull, P. B., Mattson, J., Shultz, R. & Garza, D. An instrumented mouthguard for measuring linear and angular head impact kinematics in American football (2013). 10.1007/s10439-013-0801-y PMC3954756 23604848

[21] Zhong J (2013). Finger typing driven triboelectric nanogenerator and its use for instantaneously lighting up LEDs. Nano Energy.

[22] Zhong Q (2013). A paper-based nanogenerator as a power source and active sensor. Energy Environ. Sci..

[23] Zhong Q (2015). Paper-based active tactile sensor array. Adv. Mater..

[24] Safaei M, Sodano HA, Anton SR (2019). A review of energy harvesting using piezoelectric materials: State-of-the-art a decade later (2008–2018). Smart Mater. Struct..

[25] Pastrana J (2020). Electrode effects on flexible and robust polypropylene ferroelectret devices for fully integrated energy harvesters. ACS Appl. Mater. Interfaces.

[26] Li W (2017). Nanogenerator-based dual-functional and self-powered thin patch loudspeaker or microphone for flexible electronics. Nat. Commun..

[27] Dsouza H (2020). Ferroelectret nanogenerators for loudspeaker applications: A comprehensive study. J. Sound Vibr..

[28] Cao Y, Sepúlveda N (2019). Design of flexible piezoelectric gyroscope for structural health monitoring. Appl. Phys. Lett..

[29] Cao Y (2019). Flexible ferroelectret polymer for self-powering devices and energy storage systems. ACS Appl. Mater. Interfaces.

[30] Beckwith JG, Greenwald RM, Chu JJ (2012). Measuring head kinematics in football: correlation between the head impact telemetry system and hybrid iii headform. Ann. Biomed. Eng..

[31] Walsh ES, Rousseau P, Hoshizaki TB (2011). The influence of impact location and angle on the dynamic impact response of a hybrid iii headform. Sports Eng..

[32] Sales FC, Ariati RM, Noronha VT, Ribeiro JE (2022). Mechanical characterization of PDMS with different mixing ratios. Proc. Struct. Integr..

[33] SAE J211/1–instrumentation for impact test-part 1-electronic instrumentation. *J. Soc. Automot. Eng.* (2007).

[34] Virtanen P (2020). SciPy 1.0: Fundamental Algorithms for Scientific Computing in Python. Nat. Methods.

