# Supplementary material for: Flexible, self-powered sensors for estimating human head kinematics relevant to concussions
Source: Sci Rep. 2022 Jun 23;12:8567. doi: 10.1038/s41598-022-12266-6 (PMC9226111; doi:10.1038/s41598-022-12266-6)
Supplement: Supplementary file 1 — Supplementary Information 1. [file 41598_2022_12266_MOESM1_ESM.pdf]

Supplementary material

Supplementary method 1

The role of the PDMS is to ensure there is no buckling of the FENG during compression. Given that the elastic modulus of the PDMS is higher than that of the tape, the buckling occurs at the tape and pdms protects the FENG from buckling. Also, during the tensile stress experiments, the PDMS ensures that that force is distributed throughout the area of the FENG. Prior to this working configuration, several others were tried in hopes of avoiding the buckling on the FENG. Some of which are as shown below.

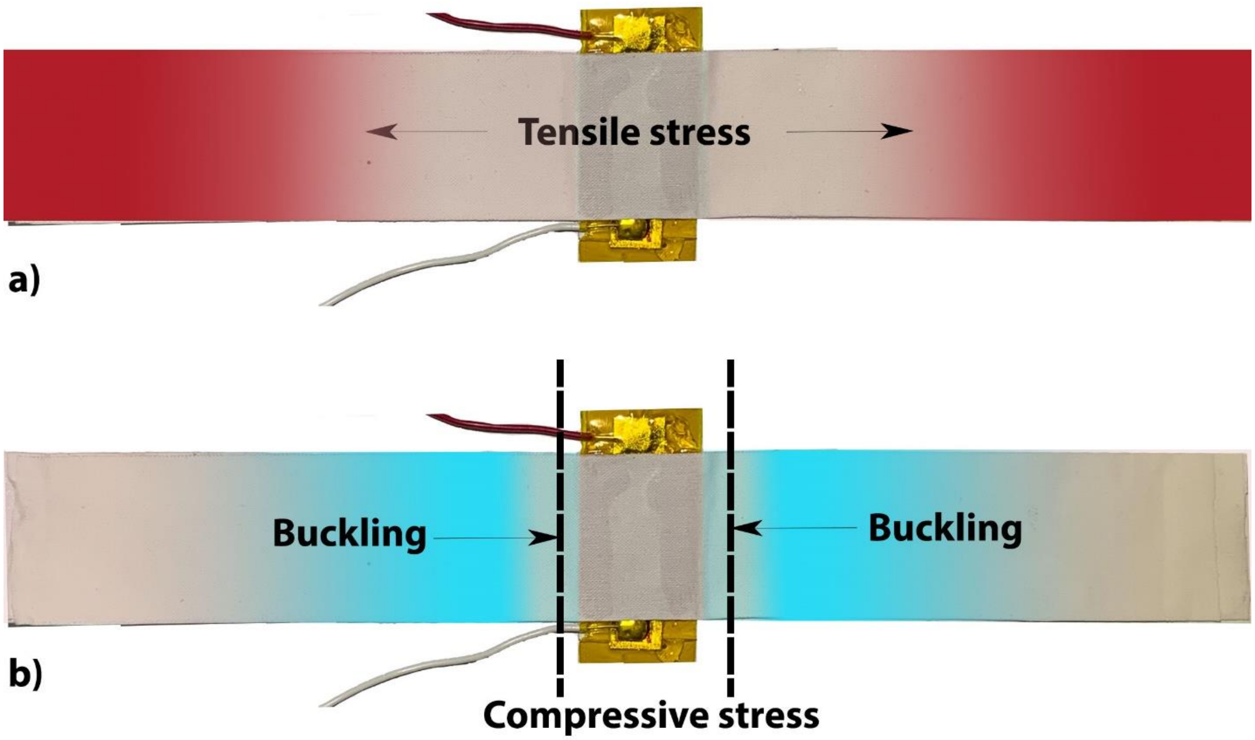

Supplementary Figure 1: a) During a tensile stress the FENG experiences deformation there by generating voltage. However during compressive stress b) the buckling at the regions indicated by the lines causes the FENG to undergo little to no deformation.

Supplementary setup 1

In this section the experimental setup is described with respect to drop towers and mounting of the neck and sensors.

The neck is connected to a U-bar using the 4 mounting holes provided and the jam nut is torqued to 1.36Nm. This jam nut is connected to the neck cable that is responsible for the stiffness of the neck. the U-bar of the other end is connected to the center plate as shown in the figure below using a single center nut. This is done so that the neck can be rotate around the z-axis (refer to figure 2 of manuscript) for future experiments.

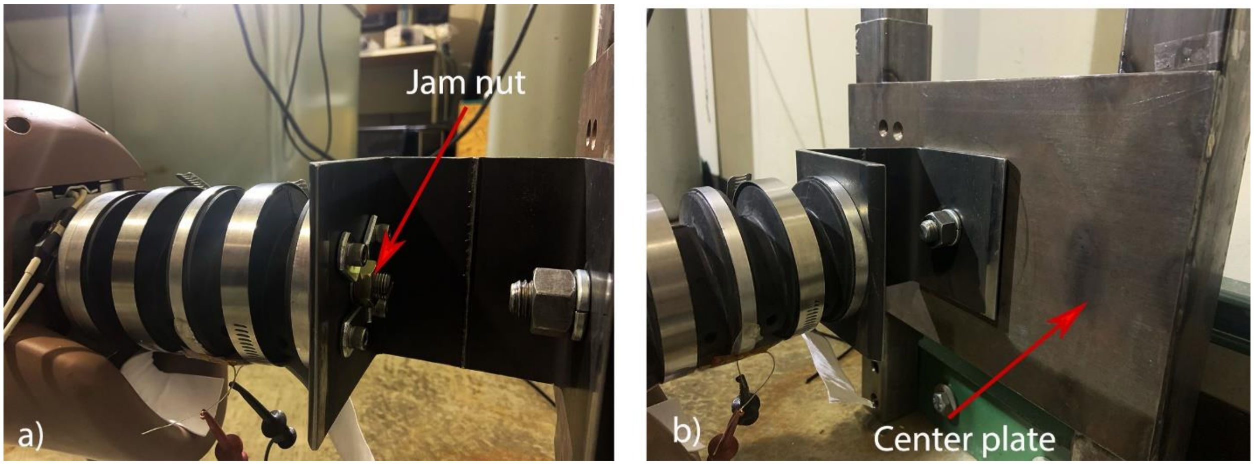

Supplementary Figure 2 : a) Dummy neck connected to the U-bar, which is then connected to the center plate as shown in b).

Supplementary discussion 1

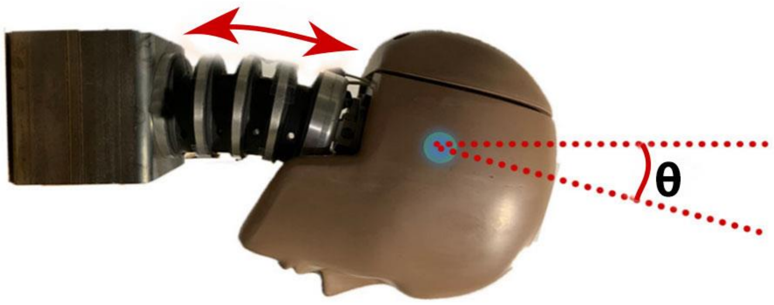

Supplementary Figure 3: Shows the Head tilt causing a flexion in the neck.

From the above figure the angular position of the head is related to tensile forces acting on the FENG (patch) placed on the neck. Assuming a simple spring model one can say that the force is proportional to position and thus velocity is proportional to the derivative of force. This is used to approximate a transfer function that is mentioned in the manuscript. The phase delay between the patch and the angular velocity observed is the result of 2 factors: 1) the inherent transfer function of the FENG (i.e. the voltage response to the applied force), and 2) the position of the patch with respect to where the sensor is placed (i.e., the head begins to rotate before the neck flexes).

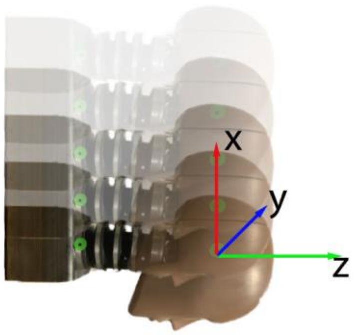

Supplementary Figure 4: Shows head experiencing only linear acceleration in x-axis.

From the above figure we can see that when the head is experiencing purely linear acceleration there is no force exerted on the FENG and hence no response.

Supplementary video: Drop Test

The supplementary video shows the drop test on the dummy head, showing how the compressive-tensile stresses are generated in the process.
